# Poor biosecurity could lead to disease outbreaks in animal populations

**DOI:** 10.1371/journal.pone.0193243

**Published:** 2018-03-07

**Authors:** Matthew J. Gray, Jennifer A. Spatz, E. Davis Carter, Christian M. Yarber, Rebecca P. Wilkes, Debra L. Miller

**Affiliations:** 1 Center for Wildlife Health, University of Tennessee Institute of Agriculture, Knoxville, Tennessee, United States of America; 2 University of Georgia Veterinary Diagnostic and Investigational Laboratory, Tifton, Georgia, United States of America; University of South Dakota, UNITED STATES

## Abstract

Human-mediated disease outbreaks due to poor biosecurity practices when processing animals in wild populations have been suspected. We tested whether not changing nitrile gloves between processing wood frog (*Lithobates sylvaticus*) tadpoles and co-housing individuals increased pathogen transmission and subsequent diseased-induced mortality caused by the emerging pathogen, ranavirus. We found that not changing gloves between processing infected and uninfected tadpoles resulted in transmission of ranavirus and increased the risk of mortality of uninfected tadpoles by 30X. Co-housing tadpoles for only 15 minutes with 10% of individuals infected resulted in ranavirus transmission and 50% mortality of uninfected tadpoles. More extreme mortality was observed when the co-housing infection prevalence was >10%. Our results illustrate that human-induced disease outbreaks due to poor biosecurity practices are possible in wild animal populations.

## Introduction

Humans can play a role in the emergence of infectious diseases in animal populations. Commonly cited examples are humans increasing stressors in the environment that compromise the immune system of a host and pathogen pollution [1, 2]. Pathogen pollution is generally defined as human-mediated translocation of a novel pathogen over large geographic distances and subsequent release into a naïve population [2]. Examples of pathogen pollution include the emergence of *Pseudogymnoascus destructans* in North American bat populations and the emergence of *Batrachochytrium salamandrivorans* in European salamander populations [3, 4].

Another way that humans can alter disease processes in animal populations is by facilitating pathogen transmission. Numerous cases exist in human medicine of iatrogenic pathogen transmission between patients and health care workers due to poor biosecurity practices [5–7]. Many pathogens are highly contagious, hence practices such as co-housing animals could result in transmission between infected and uninfected individuals [8, 9]. Some pathogens also can be shed through the skin [10, 11], hence handling different individuals without changing gloves could facilitate transmission. We tested the possibility of human-mediated pathogen transmission if gloves were not changed between handling infected and uninfected amphibians, and if infected and uninfected individuals were co-housed. The model system we used was a highly transmissible pathogen, ranavirus, that is emerging globally in ectothermic vertebrate populations [12].

## Materials and methods

We performed two experiments for this investigation: one where the researcher did not change examination gloves between handling animals and one where infected and uninfected animals were co-housed for different durations. Although biologists increasingly use examination gloves when processing animals in the wild [13, 14], there is some skepticism about using them [15] and in some cases, gloves are not changed between animals (MJG, personal observation). Also, captured animals may be co-housed. For example, amphibian tadpoles occasionally are placed together in buckets as they are processed for biological data or pathogen surveillance [16, 17]. We performed the experiments in a controlled laboratory environment at the Joe Johnson Animal Research and Teaching Unit of the University of Tennessee Institute of Agriculture. The experiments were performed using wood frog (*Lithobates sylvaticus*) larvae (tadpoles), which are known to be highly susceptible to the pathogen we used [18]. We raised the tadpoles from egg masses collected in the wild in eastern Tennessee, USA. Because the susceptibility to ranavirus can change among amphibian developmental stages [19], we began the experiments at Gosner stage 30 [20], which has been used as a standard development stage to test host susceptibility [18]. Prior to the experiment, tadpoles were raised communally in wading pools and fed commercial-grade pelleted fish food. We performed all experiments using a chimeric *Frog virus 3* (FV3)-like ranavirus originally isolated in Georgia, USA [21, 22]. Our virus replication procedures have been described previously [23].

### Glove experiment

We designed this experiment with a glove treatment (change or no change) and different infection prevalence levels. For the change treatment, we wore nitrile examination gloves and changed them between handling each individual. This treatment was crossed with two infection prevalence treatments (10 and 40%). For the no-change treatment, we wore nitrile gloves but they were not changed between handling individuals. This treatment was crossed with four infection prevalence treatments (5, 10, 20 and 40%). Each treatment was replicated five times, and each replicate consisted of processing 20 tadpoles (20 tadpoles per replicate x 5 replicates x 6 treatments) for a total of 600 tadpoles. Twenty control tadpoles were also included and experienced the identical processing procedures. Processing included handling the tadpole (mean = 11.5, SD = 3.8 seconds) and swabbing its mouthparts. Swabbing is a common nonlethal sampling technique used to test for various herpetofaunal pathogens [17, 24]. We guaranteed infection by exposing the tadpoles designated as infected to 10^3^ plaque forming units (PFU) of the FV3-like ranavirus per mL of water, which is a concentration above the Lethal Dose (LD)-50 for wood frog tadpoles [18, 25]. We exposed the tadpoles individually to the virus (10^6^ PFU total) in 2 L containers with 1 L of de-chlorinated municipal water for 72 hours. Thereafter, tadpoles were transferred to 2 L containers with 1 L of virus-free, de-chlorinated water until the experiment began three days later. Our previous work suggested that six days post-exposure to the ranavirus and dose we used would result in systemic infection in wood frog tadpoles [18]. The position of the infected tadpole during processing depended on the prevalence treatment. For the 5% treatment, the first tadpole of 20 processed was infected. For the remaining treatments, tadpoles were systematically placed with an equal number of uninfected tadpoles between them. Hence, for the 10% treatment, the first and 10^th^ tadpoles processed were infected. To minimize the possibility of contamination, three researchers handled animals: one person handled the infected tadpoles, one person handled uninfected tadpoles, and one person swabbed individuals and either changed or did not change gloves. Each tadpole was netted from its 2 L container, temporarily placed in a petri dish, and delivered to the researcher performing the swabbing. A new paper towel was placed on the work area by a fourth researcher between each tadpole within treatments, and the work surface was decontaminated with 1% Nolvasan® (Zoetis, Parsippany, NJ, USA) between each treatment [26]. After uninfected tadpoles were swabbed, they were returned to new 2 L containers with 1 L of de-chlorinated water, containers and water were changed every three days thereafter, and survival was monitored for 14 days, which is a sufficient duration for ranaviral disease to develop in wood frog tadpoles [18]. We euthanized all initially infected tadpoles after swabbing was completed and verified infection using quantitative PCR (qPCR, discussed below). Within the no-change treatment, we also swabbed gloves for evidence of shed virus after processing the last (20^th^) tadpole for each replicate. As individuals died, or at the end of the experiment, we performed necropsies, collected a homogenate of kidney and liver tissue to test for ranavirus infection using qPCR, and performed histopathology on cross-sections of the liver to verify ranaviral disease [27].

### Co-housing experiment

We designed this experiment with three co-housing duration treatments (15, 30, and 60 minutes) and three infection prevalence treatments (10%, 20%, and 40%). These treatments were crossed for a total of nine treatment combinations each replicated five times. The replicate was a 19-L bucket filled with 4 L of water, which represents typical conditions for temporarily housing tadpoles captured in the wild [16]. In each bucket, there were 10 tadpoles with the aforementioned infection percentages. For example, 2 of 10 tadpoles were infected for the 20% prevalence treatment. In many field-sampling scenarios, 10 tadpoles per bucket would represent a low co-housing density. The total number of tadpoles used in this experiment was 450 (9 treatments x 5 replicate buckets x 10 tadpoles per buckets). Infection of the tadpoles designated as infected was guaranteed following the same procedures as the glove experiment. We co-housed uninfected and infected tadpoles for 15, 30, or 60 minutes, which is a reasonable range of co-housing duration when sampling in the wild. We clipped a small section of tail from the infected tadpoles prior to co-housing so they could be identified. After the co-housing duration, we euthanized all initially infected tadpoles and verified infection using qPCR. The uninfected co-housed tadpoles were removed from the buckets with individual nets and placed in individual 2 L containers with 1 L of de-chlorinated municipal water, and their survival followed for 14 days. As individuals died and at the end of the experiment, we necropsied tadpoles, used a homogenate of liver and kidney tissue to test for ranavirus infection, and performed histopathology on liver samples to confirm ranaviral disease. We also monitored 10 control tadpoles (one replicate bucket) exposed to the same co-housing conditions and verified no infection with qPCR.

### Pathogen testing

Our goal with pathogen testing was to verify that ranavirus infection was associated with the observed mortality events. Because a large number of tadpoles was used in this study, we tested three of five random replicates per treatment per experiment, which was 360 and 270 tadpoles for the glove and co-housing experiments, respectively. We extracted genomic DNA from the homogenate of liver and kidney tissue using a DNeasy Blood and Tissue Kit (Qiagen, Hilden, Germany). Prior to qPCR analysis, we eluted 100 μl of the extracted DNA and quantified the amount of DNA present in each sample. We used a model ABI 7900HT Fast Real-Time PCR System (Life Technologies, Carlsbad, California, USA) to test samples for ranavirus DNA using PCR primers and probe targeting a 70-bp region of the ranavirus major capsid protein [28, 29]. We considered a sample infected if the qPCR cycle threshold (CT) value was less than 32 based on standardized optimization with known quantities of ranavirus. For each qPCR analysis, we ran each extracted DNA sample in duplicate along with 2 positive controls (i.e. positive viral DNA and viral DNA from a ranavirus-positive amphibian) and 2 negative controls (i.e. DNA from a ranavirus negative amphibian and a sample containing only molecular grade water [27]). Survival and qPCR data for the glove and co-housing experiments are provided in the S1 Dataset and S2 Dataset files, respectively.

### Histopathology

A different set of autoclaved instruments was used for each animal necropsied. After collection of liver and kidney samples for PCR analysis, the remaining animal was placed into 10% neutral buffered formalin. Formalin-fixed liver tissue was routinely processed for histopathology at the University of Tennessee Veterinary Medical Center Diagnostic Laboratory, embedded in paraffin blocks, sectioned at approximately 5 μm onto glass slides, which were then stained with Hematoxylin and Eosin and examined by light microscopy for evidence of ranaviral disease [27].

### Data presentation and statistical analyses

We estimated survival functions for all treatments using the Kaplan-Meier method, and used a log-rank test to determine if statistical differences (α = 0.05) existed among survival curves [30]. When differences existed, we estimated hazard ratios (i.e., instantaneous rate of death) for each treatment as an index of mortality risk using Cox’s proportional hazards model [31, 32]. All survival analyses were performed using R (v. 3.3) statistical software (https://www.r-project.org). Code for the survival analyses is provided in S1 Code and S2 Code files for the glove and co-housing experiments, respectively. We reported infection prevalence results by treatment according to the fate of individuals (clinical infection = died, infected; subclinical infection = survived, infected; incidental mortality = died, uninfected; and resistant = survived, uninfected). We also reported mean viral loads on the gloves that were not changed for each of the infection prevalence treatments, and tested for differences using a one-way analysis-of-variance.

### Ethics statement

All husbandry and euthanasia procedures described herein were in accordance with the Association for Assessment and Accreditation of Laboratory Animal Care International Standards and followed recommendations provided in the Amphibian Husbandry Resource Guide of the Association of Zoos and Aquariums and the Guide for Euthanasia published by the American Veterinary Medical Association. All activities were approved by the Institutional Animal Care and Use Committee (IACUC) at the University of Tennessee-Knoxville (UTK, protocol #2357). Collection of egg masses from the wild was approved under Tennessee Wildlife Resources Agency Scientific Collection Permit #1990 and followed collection and transport protocols described in approved UTK IACUC protocol #2357.

## Results

### Glove experiment

**Fig 1:**
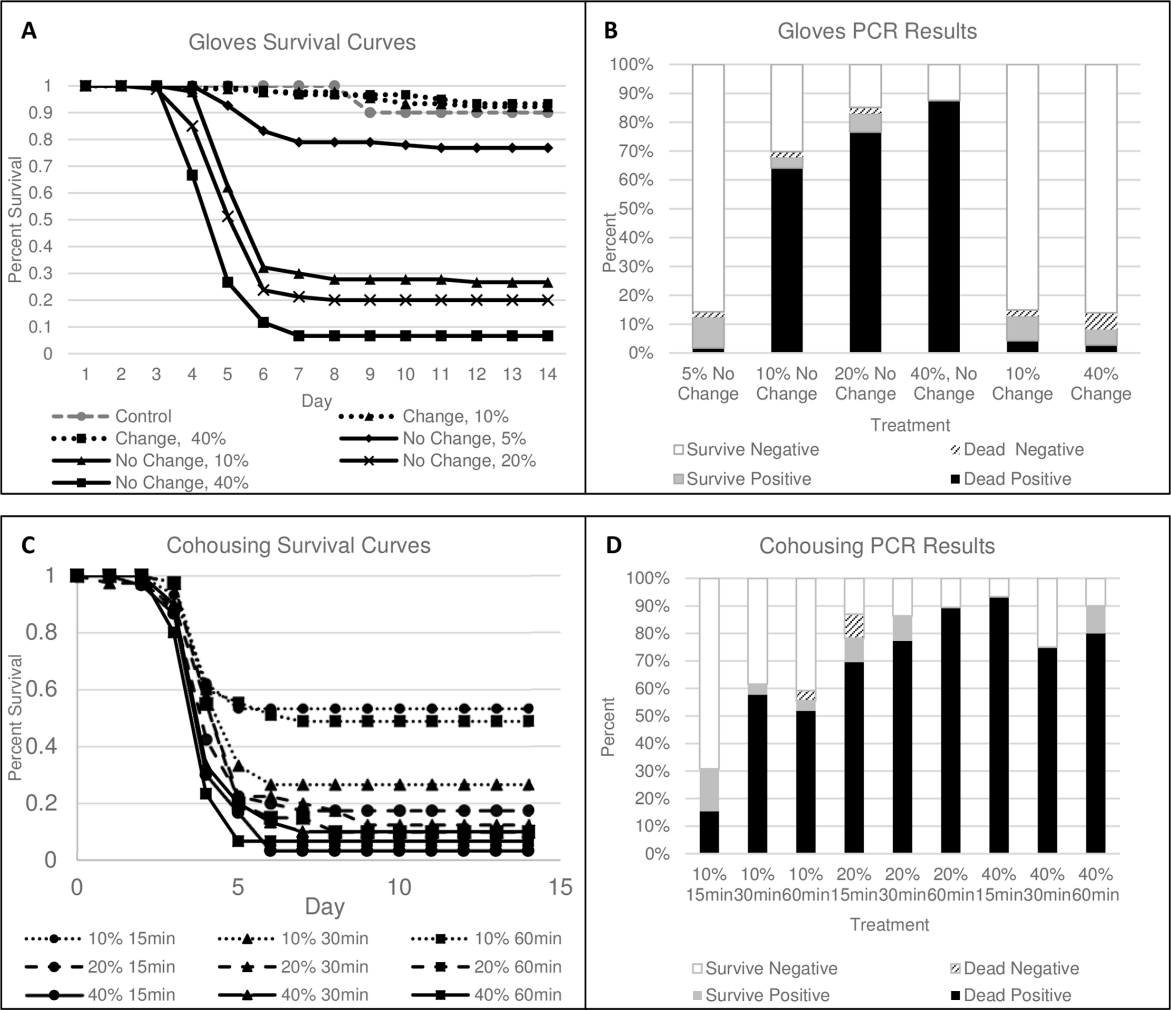
(A) Survival of uninfected wood frog (*Lithobates sylvaticus*) tadpoles that were processed with infected tadpoles at different levels of infection prevalence with and without changing gloves, (B) corresponding mortality and infection prevalence for 3 of 5 replicates in (A), (C) survival of uninfected wood frog tadpoles that were co-housed with infected tadpoles at different levels of infection prevalence and for different durations, (D) corresponding mortality and infection prevalence for 3 of 5 replicates in (C).

**Fig 2:**
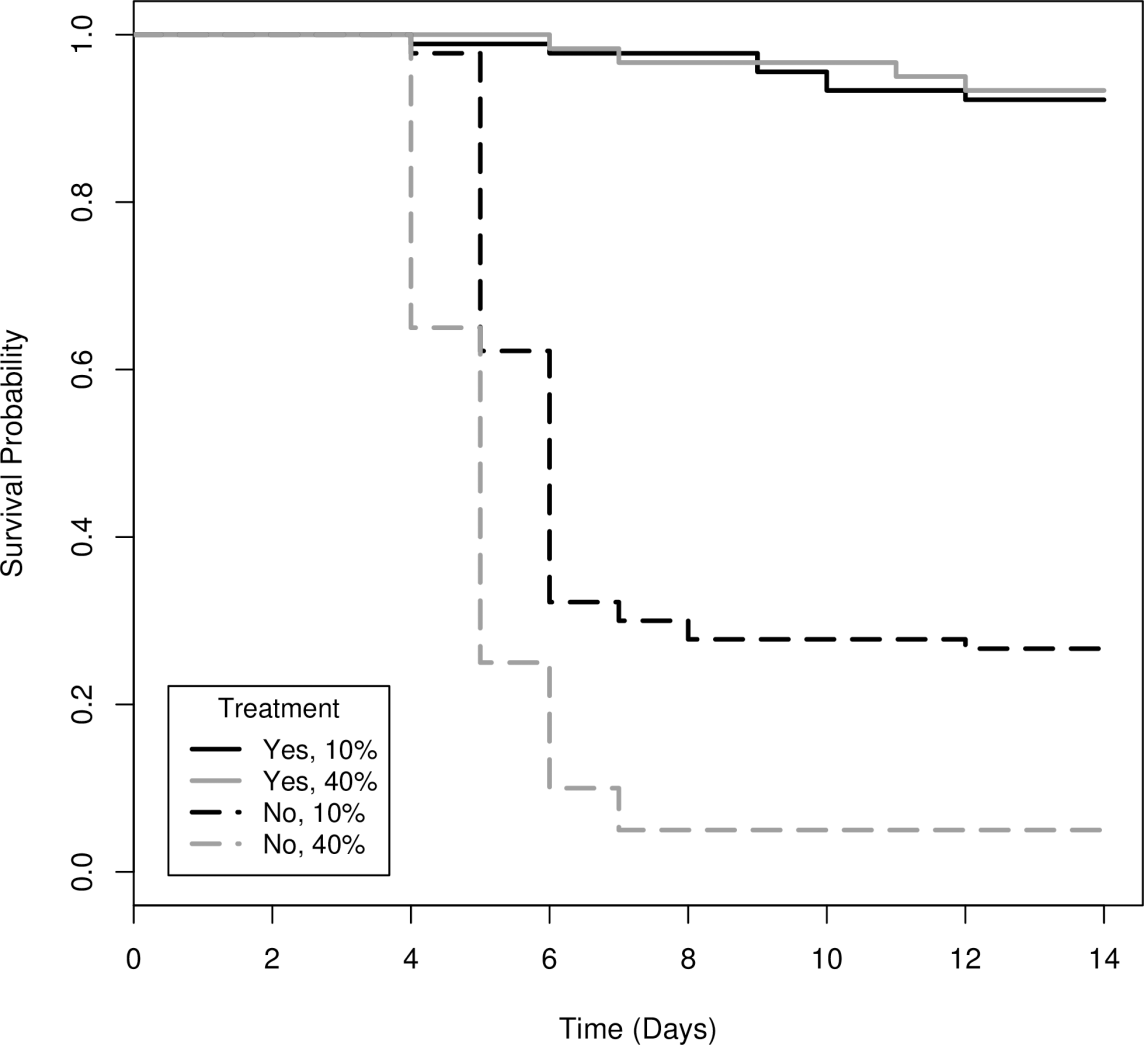
Survival functions for uninfected wood frog (*Lithobates sylvaticus*) tadpoles that were processed with and without changing gloves (yes, no) at two known ranavirus infection prevalence levels (10%, 40%).

**Fig 3:**
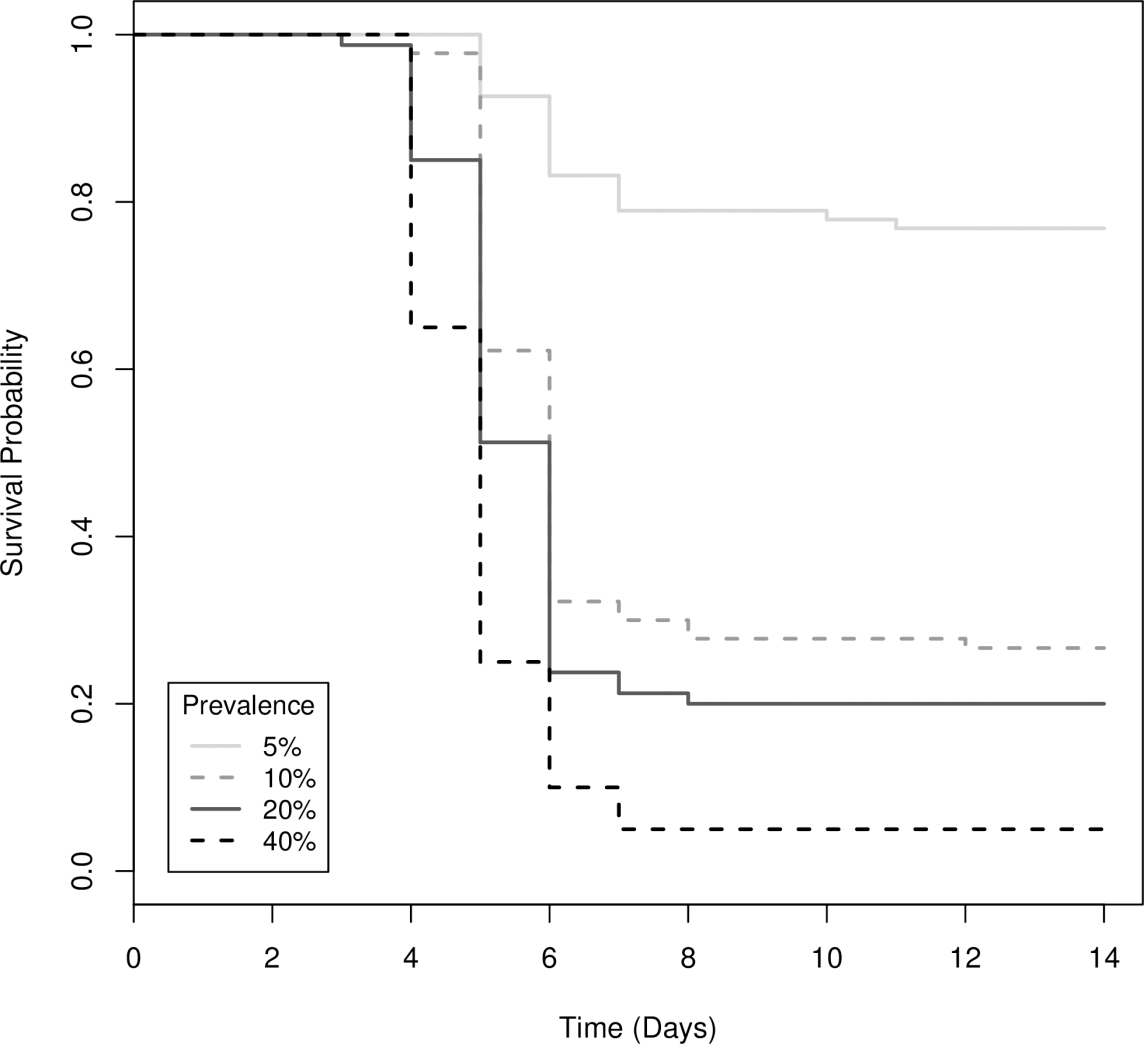
Survival functions for uninfected wood frog (*Lithobates sylvaticus*) tadpoles that were processed without changing gloves at four known ranavirus infection prevalence levels.

Survival differed significantly among the glove treatments (Χ^2^_(6)_ = 328, *P* < 0.001; Fig 1A). Survival of control animals did not differ from the glove change treatment (Χ^2^_(1)_ = 0.2, *P* = 0.68), hence controls were removed from further analyses to allow direct comparison between change-no change treatments. Not changing gloves increased the risk of mortality of uninfected tadpoles by nearly 30X compared to changing gloves between animals (Table 1, Fig 2). Increasing the prevalence of infected individuals processed from 10% to 40% increased the risk of mortality of uninfected tadpoles by 2.4X (Table 1, Fig 2). However, if gloves were not changed, increasing the prevalence of infected individuals processed from 5–40% increased the risk of mortality of uninfected individuals by 5 – 13X (Table 2, Fig 3).

**Table 1:** The difference in the instantaneous rate of mortality (i.e., hazard ratio) for uninfected wood frog (*Lithobates sylvaticus*) tadpoles that were processed with and without changing gloves (yes vs. no) at two known ranavirus infection prevalence levels (10 vs. 40%).

Treatment		Coefficient: b	Hazard Ratio (HR): exp(b)	95% CI for HR	*P*-value
Glove Change	Yes	0	1	-	-
	No	3.383	29.465	(15.674, 55.389)	<0.001
Prevalence	10%	0	1	-	-
	40%	0.889	2.433	(1.719, 3.444)	<0.001

**Table 2:** The difference in the instantaneous rate of mortality (i.e., hazard ratio) for uninfected wood frog (*Lithobates sylvaticus*) tadpoles that were processed without changing gloves at four known ranavirus infection prevalence levels.

Prevalence	Coefficient: b	Hazard Ratio (HR): exp(b)	95% CI for HR	*P*-value
5%	0	1	-	-
10%	1.597	4.940	(3.042, 8.023)	<0.001
20%	1.872	6.502	(3.992, 10.589)	<0.001
40%	2.553	12.847	(7.784, 21.203)	<0.001

**Fig 4:**
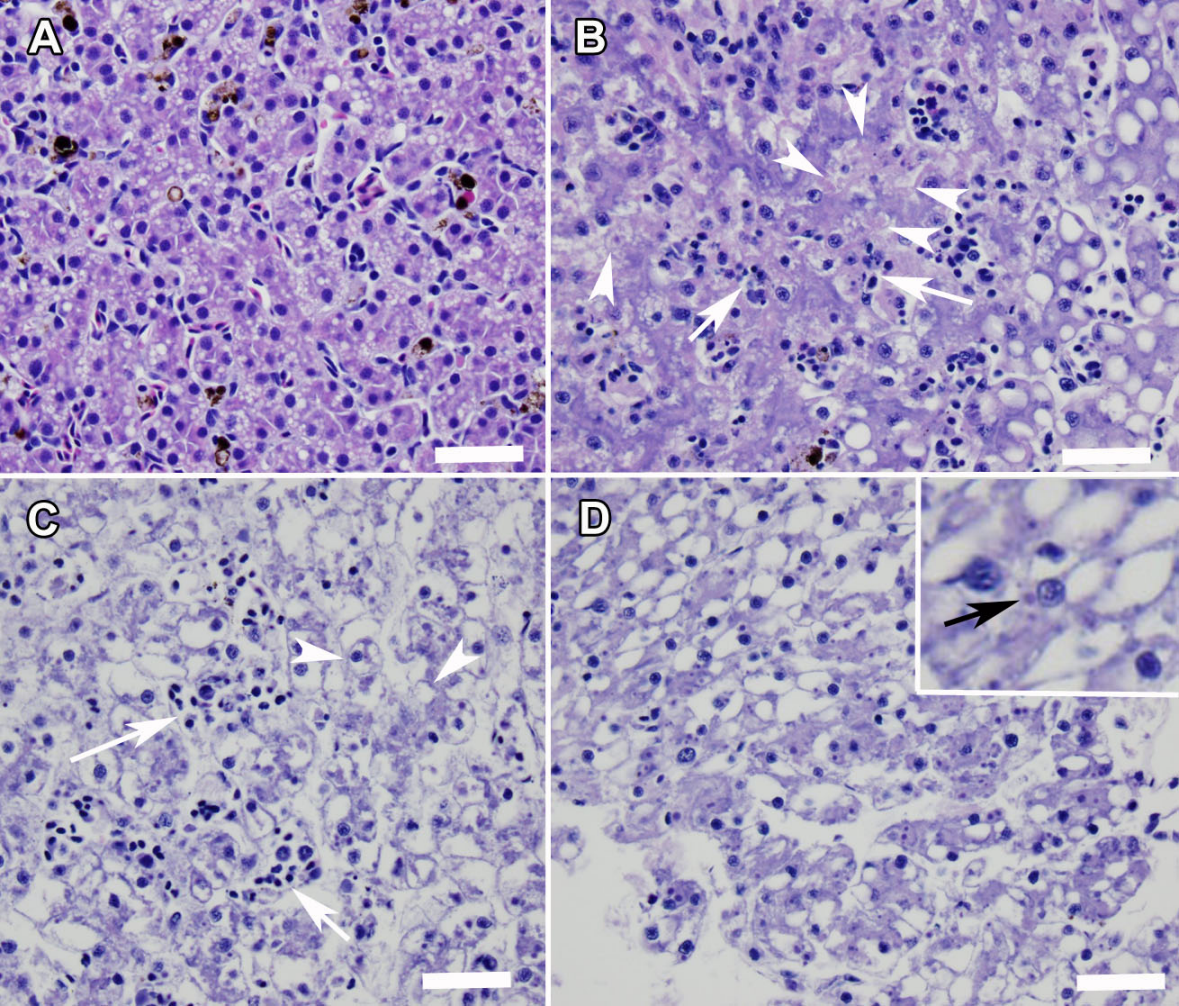
Sections of liver from a control animal (A) and from ranavirus qPCR positive animals demonstrating ranaviral disease (B-D). (B) Necrosis of hematopoietic cells (arrows) and degeneration and necrosis of hepatocytes (arrowheads) in a liver from an amphibian co-housed for 60 minutes in a container where 40% of the amphibians were infected with ranavirus. (C) Diffuse necrosis of hematopoietic cells (arrows) and hepatocytes (arrowheads) in a liver from an amphibian processed in a simulated swabbing event where 10% of the amphibians were infected with ranavirus and gloves were not changed during processing. (D) Intracytoplasmic inclusion bodies (inset) and diffuse necrosis of hematopoietic cells and hepatocytes in a liver from an amphibian processed in a simulated swabbing event where 40% of the amphibians were infected with ranavirus and gloves were not changed during processing. Hematoxylin and Eosin stain. Bar equals 50 μm.

**Fig 5:**
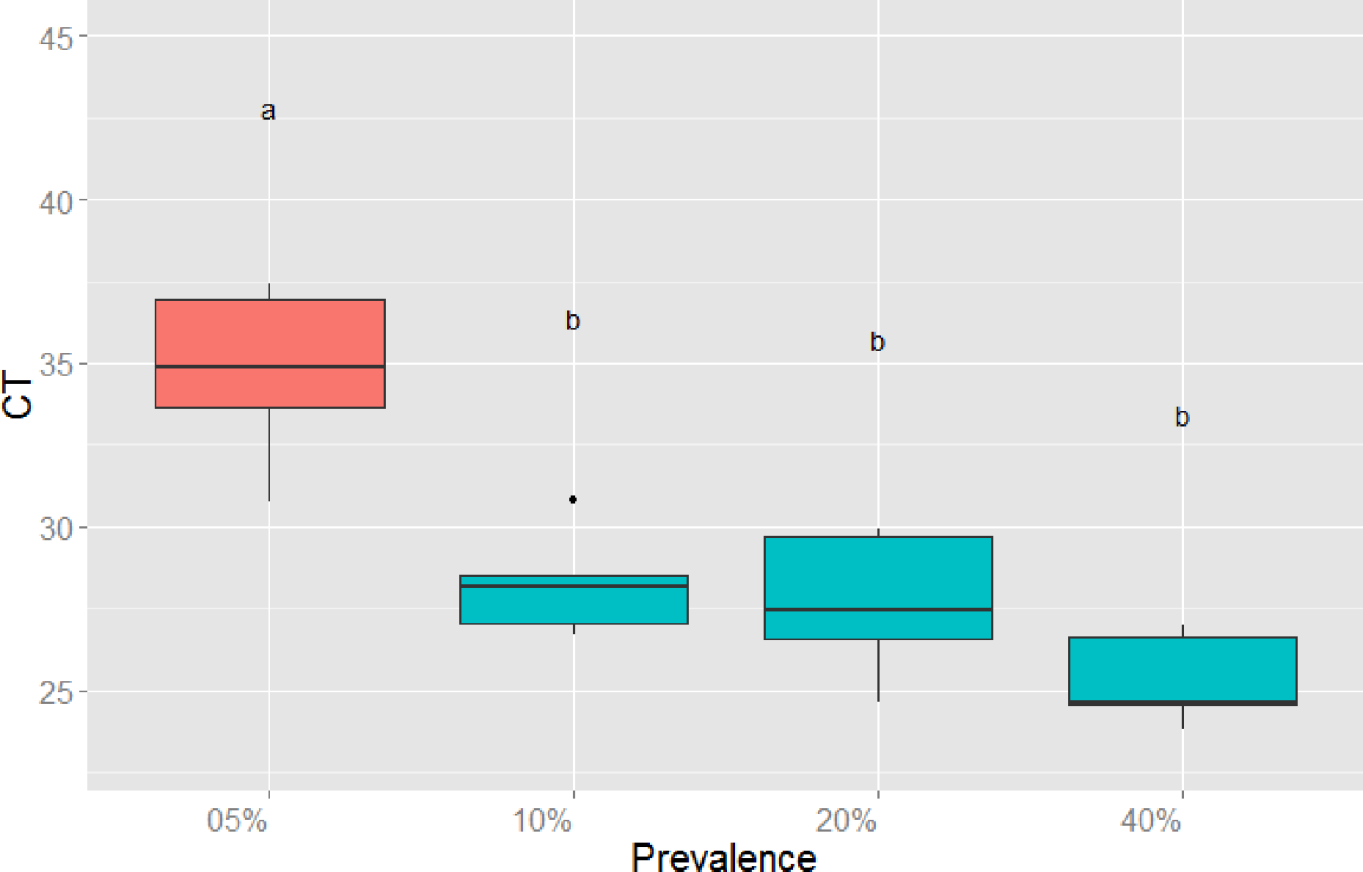
Cycle threshold (CT) for qPCR on swabs taken from gloves at four known ranavirus prevalence levels after infected wood frog (*Lithobates sylvaticus*) tadpoles were processed. Low CT corresponds to higher viral load; different letters above boxes indicate significant differences by ANOVA and Tukey’s post-hoc comparison test.

Quantitative PCR verified that 85% of infected individuals died and 15% survived with subclinical infections after 14 days (Fig 1B). Ranaviral disease, including liver necrosis and viral inclusion bodies, was confirmed by histopathology (Fig 4). For the no-change treatment, significantly more virus occurred on gloves when >5% of individuals that were processed were infected (Fig 5). A small fraction (2%) of tadpoles that were processed died with no detectable infection (Fig 1B). Also, two control tadpoles died, but they were not infected. Interestingly, some transmission occurred in the glove-change treatments (11% infection summed across both treatments), resulting in approximately 7% total mortality (i.e., sum of black bars across change treatments in Fig 1B). All tadpoles that were exposed to ranavirus that we processed as the infected individuals were infected with ranavirus and contained high viral loads in their livers and kidneys (mean = 1,569, SE = 252 PFU per 0.25 μg of gDNA).

### Co-housing experiment

**Fig 6:**
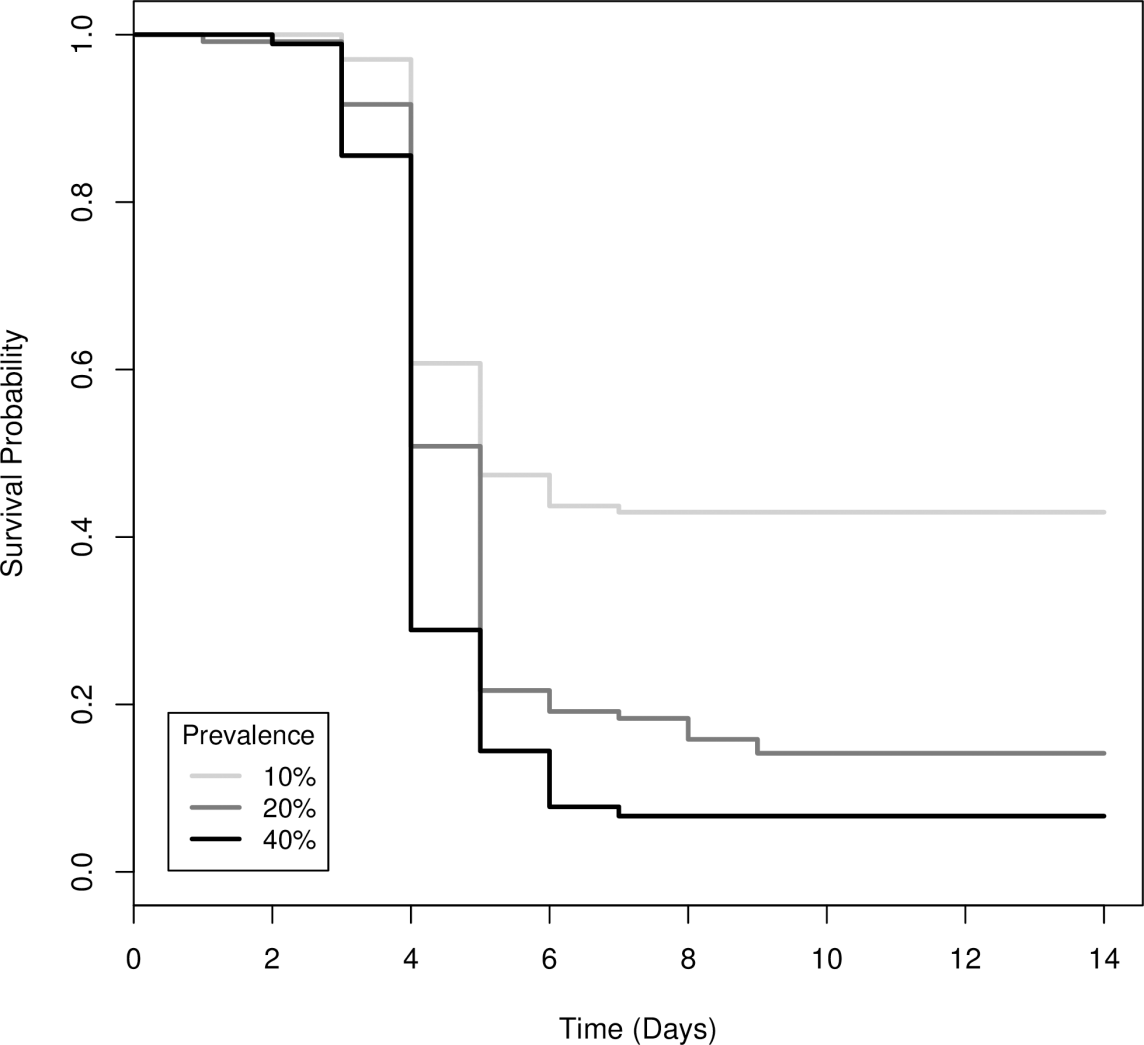
Survival functions for uninfected wood frog (*Lithobates sylvaticus*) tadpoles that were co-housed with infected tadpoles at three known ranavirus infection prevalence levels.

Survival differed significantly among the co-housing treatments (Χ^2^_(8)_ = 57, *P* < 0.001; Fig 1C). Co-housing 10 tadpoles for 15, 30 or 60 minutes when only one individual was infected resulted in 50–75% mortality of uninfected tadpoles after 14 days (Fig 1C). If two of 10 tadpoles were infected, average mortality of uninfected tadpoles among co-housing durations was 86%. If 40% of tadpoles were infected, average mortality of uninfected tadpoles was 93% (Fig 1C). The risk of mortality for uninfected individuals doubled and tripled if 20% and 40% of co-housed tadpoles were infected, respectively, compared to when only 10% were infected (Table 3, Fig 6). There was no difference in risk of mortality among co-housing durations (Table 3).

**Table 3:** The difference in the instantaneous rate of mortality (i.e., hazard ratio) for uninfected wood frog (*Lithobates sylvaticus*) tadpoles that were co-housed with infected tadpoles at three durations and three known ranavirus infection prevalence levels.

Treatment		Coefficient: b	Hazard Ratio (HR): exp(b)	95% CI for HR	*P*-value
Prevalence	10%	0	1	-	-
	20%	0.665	1.945	(1.445, 2.619)	<0.001
	40%	1.058	2.882	(2.104, 3.947)	<0.001
Duration	15 min	0	1	-	-
	30 min	0.118	1.125	(0.837, 1.513)	0.434
	60 min	0.073	1.076	(0.796, 1.456)	0.634

Quantitative PCR verified that 91% of infected individuals died and 8% survived with subclinical infections after 14 days (Fig 1D). Ranaviral disease, including liver necrosis and viral inclusion bodies, was confirmed by histopathology (Fig 4). A small fraction (1%) of co-housed individuals died with no detectable infection (Fig 1D), and no control animals died or were infected. All tadpoles that were exposed to ranavirus that we used as the infected individuals in the co-housing buckets were infected with ranavirus and contained high viral loads in their livers and kidneys (mean = 26,933 SE = 1,837 PFU per 0.25 μg of gDNA).

## Discussion

Our study demonstrates that poor biosecurity practices can increase pathogen transmission and disease-related mortality in wild amphibians that are processed as part of routine epidemiological and biological studies. Not changing gloves between processing infected amphibians increased the risk of mortality for uninfected individuals by 30X. Some ranavirus transmission and mortality (~7%) also occurred if gloves were changed between animals, illustrating that even under controlled laboratory conditions there is a risk of transmitting pathogens among processed individuals. Co-housing infected amphibians with uninfected individuals at low densities for 15–60 minutes increased disease-related mortality by 50–95%. Collectively, these results emphasize the importance of good biosecurity practices when processing amphibians in the field or laboratory if pathogens are present.

Several researchers that study amphibians have advocated single glove use for each processed animal [17, 33, 34]; however, biologists have been reluctant to change gloves between animals due to the cost or because of waste generated [13]. In addition, some examination gloves appear to have anti-microbial properties against certain pathogens. For example, the fungus, *Batrachochytrium dendrobatidis*, was inactivated on nitrile and vinyl gloves in <1 min; however, this effect was reduced if gloves were rinsed with water or if latex or polyurethane gloves were used [13]. Although the nitrile gloves used in our experiment may have had anti-viral properties, clearly sufficient virus remained on them to facilitate transmission to a large percentage of tadpoles that were processed. In addition, virus was detected on our gloves using qPCR after processing individuals. Not changing gloves might also contribute to sample contamination and increase false positive qPCR test results [35].

Use of bare hands has been suggested as an alternative to gloves, because human hands have natural antimicrobial properties and their temperature may facilitate inactivation of some pathogens [13]. However, the persistence of pathogens on bare hands is longer than on examination gloves [13, 36, 37], and gloves can protect researchers from pathogens in the environment or on animals that are zoonotic [33]. Bare hands also may affect sample quality. For example, quality RNA in amphibian mucosome was greater in samples when animals were processed with gloves compared to bare hands [38].

Our results also indicate that changing gloves between each animal can still result in occasional pathogen transmission. Several human medicine studies have documented that hands and clothes of health care workers can become contaminated even though gowns are worn and gloves are changed frequently [39]. Double gloving has been shown to reduce the likelihood of pathogen transmission during laryngoscopy and intubation [40]. Gray et al. [17] suggested that a disposable plastic bag wrapped around a gloved hand might function similar to a double glove.

Dipping gloves into disinfectant between processing animals or wearing gloves with manufacture-coated disinfectants might reduce iatrogenic pathogen transmission [17, 41, 42]; however, these practices may have toxic effects on wild animals. Rinsing gloves with distilled water prior to use can reduce the toxic effects of gloves on amphibians [14], but it also reduces their antimicrobial properties. Cashins et al. [43] warned about possible toxic effects of gloves on amphibians; however, this team of researchers later published that the benefits of using gloves exceeded possible negative effects [14]. Indeed, we observed some control mortality (8%) in our experiment possibly associated with processing, but the risk of mortality by accidental ranavirus transmission was about 30X greater if gloves were not changed. Gray et al. [17] recommended changing gloves between processing herpetofauna, which our data support.

Co-housing uninfected and infected tadpoles also resulted in substantial transmission of ranavirus and increased mortality over control animals by 50–93%. Fifteen minutes was sufficient to increase mortality by 50–75%. Isolation is standard procedure for animals under quarantine or in captivity with unknown infection status [44]; however, this procedure has been used less frequently in the wild. Gray et al. [17] provided several recommendations for how to isolate herpetofaunal species to prevent iatrogenic pathogen transmission during biological processing.

Our results illustrate a clear, negative impact on amphibians that are processed using poor biosecurity practices; however, it is unknown whether releasing these animals into the wild would manifest into a disease outbreak in a population. Disease outcomes likely would be a consequence of pathogen virulence, host density, and environmental conditions [45]. For FV3-like ranaviruses in wood frog populations, it is reasonable to foresee a subsequent outbreak, because this species is very susceptible to ranavirus [18], the tadpoles exist at high densities [46], and the pathogen can be transmitted via direct contact, exposure to contaminated water or sediment, and by necrophagy [11, 47]. Additionally, environmental persistence of FV3-like ranaviruses outside the host is probably at least one week [48, 49]. It has been casually suggested that strict biosecurity practices are unnecessary if a pathogen already exists in a wild animal population (MJG, personal observation). In general, we disagree with this recommendation, because in many cases, processing animals increases the probability of pathogen transmission compared to endemic levels, which could facilitate the start of an epidemic in the wild. As responsible scientists and natural resource practitioners, we should exercise prudent biosecurity practices that result in minimal impacts on the animal populations we study [17].

## Supporting information

S1 Dataset Data (.csv) for glove experiment. (CSV)

S2 Dataset Data (.csv) for co-housing experiment. (CSV)

S1 Code R code for survival analyses; glove experiment. (R)

S2 Code R code for survival analyses; co-housing experiment. (R)

## References

[1] Rollins-Smith LA. Amphibian immunity–stress, disease, and climate change. Dev Comp Immunol. 2017;66:111–9. 10.1016/j.dci.2016.07.002. 27387153

[2] Cunningham AA, Daszak P, Rodriguez JP. Pathogen pollution: Defining a parasitological threat to biodiversity conservation. J Parisitol. 2003;89 (suppl):S78–S83.

[3] Martel A, Blooi M, Adriaensen C, Van Rooij P, Beukema W, Fisher MC, et al Recent introduction of a chytrid fungus endangers Western Palearctic salamanders. Science. 2014;346(6209):630–1. 10.1126/science.1258268 WOS:000343799700048. 25359973 PMC5769814

[4] Leopardi S, Blake D, Puechmaille SJ. White-Nose Syndrome fungus introduced from Europe to North America. Curr Biol. 2015;25(6):R217–9. Epub 2015/03/19. 10.1016/j.cub.2015.01.047 . 25784035

[5] Tarantola A, Abiteboul D, Rachline A. Infection risks following accidental exposure to blood or body fluids in health care workers: a review of pathogens transmitted in published cases. Am J Infect Control, 2006;34(6):367–75. Epub 2006/08/01. 10.1016/j.ajic.2004.11.011 . 16877106 PMC7115312

[6] Pedrosa PB, Cardoso TA. Viral infections in workers in hospital and research laboratory settings: a comparative review of infection modes and respective biosafety aspects. Int J Infect Dis. 2011;15(6):e366–76. Epub 2011/04/19. 10.1016/j.ijid.2011.03.005 . 21497126 PMC7110847

[7] Ye D, Shan J, Huang Y, Li J, Li C, Liu X, et al A gloves-associated outbreak of imipenem-resistant *Acinetobacter baumannii* in an intensive care unit in Guangdong, China. BMC Infect Dis. 2015;15:179 Epub 2015/04/18. 10.1186/s12879-015-0917-9 ; PubMed Central PMCID: PMCPmc4415246. 25886493 PMC4415246

[8] Officer K, Lan NT, Wicker L, Hoa NT, Weegenaar A, Robinson J, et al Foot-and-mouth disease in Asiatic black bears (*Ursus thibetanus*). J Vet Diagn Invest. 2014;26(5):705–13. Epub 2014/08/20. 10.1177/1040638714547256 . 25135011

[9] Gerhold R, Hickling G. Diseases associated with translocation of captive cervids in North America. Wildl Soc Bull. 2016;40(1):25–31. 10.1002/wsb.638

[10] Kilpatrick AM, Briggs CJ, Daszak P. The ecology and impact of chytridiomycosis: an emerging disease of amphibians. Trends Ecol Evol. 2010;25(2):109–18. Epub 2009/10/20. 10.1016/j.tree.2009.07.011 . 19836101

[11] Brunner JL, Storfer A, Gray MJ, Hoverman JT. Ranavirus Ecology and Evolution: From Epidemiology to Extinction In: Gray MJ, Chinchar VG, editors. Ranaviruses: Lethal Pathogens of Ectothermic Vertebrates. Springer International Publishing; 2015 p. 71–104. 10.1007/978-3-319-13755-1

[12] Gray MJ, Chinchar VG, editors. Ranaviruses: Lethal Pathogens of Ectothermic Vertebrates. 1 ed: Springer International Publishing; 2015 10.1007/978-3-319-13755-1

[13] Mendez D, Webb R, Berger L, Speare R. Survival of the amphibian chytrid fungus *Batrachochytrium dendrobatidis* on bare hands and gloves: hygiene implications for amphibian handling. Dis Aquat Organ. 2008;82(2):97–104. Epub 2009/01/20. 10.3354/dao01975 . 19149372

[14] Greer A, Schock Brunner J, Johnson Picco, Cashins S, et al Guidelines for the safe use of disposable gloves with amphibian larvae in light of pathogens and possible toxic effects. Herpetol Rev. 2009; 40(2):145–7.

[15] Gutleb AC, Bronkhorst M, van den Berg JH, Murk AJ. Latex laboratory-gloves: an unexpected pitfall in amphibian toxicity assays with tadpoles. Environ Toxicol Pharmacol. 2001;10(3):119–21. Epub 2001/07/01. . 21782566 10.1016/s1382-6689(01)00091-6

[16] Gray MJ, Chamberlain MJ, Buehler DA, Sutton WB. Wetland Wildlife Monitoring and Assessment In: Anderson JT, Davis CA, editors. Wetland Techniques. 2. Secaucus, New Jersey, USA: Springer; 2013 p. 265–318

[17] Gray MJ, Duffus A, Haman KH, Harris R, Allender M, Thompson TA, et al Pathogen surveillance in herpetofaunal populations: guidance on study design, sample collection, biosecurity, and intervention strategies. Herpetol Rev. 2017; 48:334–51.

[18] Hoverman JT, Gray MJ, Haislip NA, Miller DL. Phylogeny, Life History, and Ecology Contribute to Differences in Amphibian Susceptibility to Ranaviruses. EcoHealth. 2011;8(3):301–19. 10.1007/s10393-011-0717-7 WOS:000301184200007. 22071720

[19] Haislip NA, Gray MJ, Hoverman JT, Miller DL. Development and Disease: How Susceptibility to an Emerging Pathogen Changes through Anuran Development. PLoS One. 2011;6(7):6 10.1371/journal.pone.0022307 WOS:000293097300030. 21799820 PMC3142128

[20] Gosner KL. A simplified table for staging anuran embryos and larvae with notes on identification. Herpetologica. 1960;16:183–90. ZOOREC:ZOOR09700000530.

[21] Miller DL, Rajeev S, Gray MJ, Baldwin CA. Frog Virus 3 Infection, Cultured American Bullfrogs. Emerging Infect Dis. 2007;13(2):342–3. 10.3201/eid1302.061073 PMC2725843. 17479910 PMC2725843

[22] Claytor SC, Subramaniam K, Landrau-Giovannetti N, Chinchar VG, Gray MJ, Miller DL, et al Ranavirus phylogenomics: signatures of recombination and inversions among bullfrog ranaculture isolates. Virology. 2017;511:330–343. 10.1016/j.virol.2017.07.028 28803676

[23] Hoverman JT, Gray MJ, Miller DL. Anuran susceptibilities to ranaviruses: role of species identity, exposure route, and a novel virus isolate. Dis Aquat Organ. 2010;89(2):97–107. Epub 2010/04/21. 10.3354/dao02200 . 20402227

[24] Retallick RW, Miera V, Richards KL, Field KJ, Collins JP. A non-lethal technique for detecting the chytrid fungus *Batrachochytrium dendrobatidis* on tadpoles. Dis Aquat Organ. 2006;72(1):77–85. Epub 2006/10/28. 10.3354/dao072077 . 17067076

[25] Warne RW, Crespi EJ, Brunner JL. Escape from the pond: stress and developmental responses to ranavirus infection in wood frog tadpoles. Funct Ecol. 2011;25(1):139–46. 10.1111/j.1365-2435.2010.01793.x

[26] Bryan LK, Baldwin CA, Gray MJ, Miller DL. Efficacy of select disinfectants at inactivating Ranavirus. Dis Aquat Organ. 2009;84(2):89–94. Epub 2009/05/30. 10.3354/dao02036 . 19476278

[27] Miller DL, Pessier AP, Hick P, Whittington RJ. Comparative Pathology of Ranaviruses and Diagnostic Techniques In: Gray MJ, Chinchar VG, editors. Ranaviruses: Lethal Pathogens of Ectothermic Vertebrates. Springer International Publishing; 2015 p. 171–208. 10.1007/978-3-319-13755-1

[28] Gray MJ, Miller DL, Hoverman JT. Reliability of non-lethal surveillance methods for detecting ranavirus infection. Dis Aquat Org. 2012;99(1):1–6. 10.3354/dao02436 WOS:000304049000001. 22585297

[29] Picco AM, Brunner JL, Collins JP. Susceptibility of the endangered California tiger salamander, *Ambystoma californiense*, to Ranavirus infection. J Wildl Dis. 2007;43(2):286–90. WOS:000247129600015. 10.7589/0090-3558-43.2.286 17495315

[30] Jager KJ, van Dijk PC, Zoccali C, Dekker FW. The analysis of survival data: the Kaplan-Meier method. Kidney Int. 2008;74(5):560–5. Epub 2008/07/04. 10.1038/ki.2008.217 . 18596735

[31] Cox DO D.R. Analysis of Survival Data: Chapman and Hall; 1984. 212 p.

[32] Gray MJ, Brunner JL, Earl JE, Ariel E. Design and Analysis of Ranavirus Studies: Surveillance and Assessing Risk In: Gray MJ, Chinchar VG, editors. Ranaviruses: Lethal Pathogens of Ectothermic Vertebrates. Springer International Publishing; 2015 p. 209–40. 10.1007/978-3-319-13755-1

[33] Phillott AD, Speare R, Hines HB, Skerratt LF, Meyer E, McDonald KR, et al Minimising exposure of amphibians to pathogens during field studies. Dis Aquat Organ. 2010;92(2–3):175–85. Epub 2011/01/29. 10.3354/dao02162 . 21268979

[34] Schmeller DS, Loyau A, Dejean T, Miaud C. Using amphibians in laboratory studies: precautions against the emerging infectious disease chytridiomycosis. Lab Anim. 2011;45(1):25–30. Epub 2010/11/16. 10.1258/la.2010.010101 . 21075827

[35] Skerratt LF, Mendez D, McDonald KR, Garland S, Livingstone J, Berger L, et al Validation of Diagnostic Tests in Wildlife: The Case of Chytridiomycosis in Wild Amphibians. J Herpetol. 2011;45(4):444–50. WOS:000305873900008.

[36] Greene C, Vadlamudi G, Eisenberg M, Foxman B, Koopman J, Xi C. Fomite-fingerpad transfer efficiency (pick-up and deposit) of *Acinetobacter baumannii*—with and without a latex glove. Am J Infect Control. 2015;43(9):928–34. 10.1016/j.ajic.2015.05.008. 26141689 PMC10062061

[37] Monaghan JM, Hutchison ML. Ineffective hand washing and the contamination of carrots after using a field latrine. Lett Appl Microbiol. 2016;62(4):299–303. Epub 2016/01/23. 10.1111/lam.12549 . 26797849

[38] Robertson LS, Fellers GM, Marranca JM, Kleeman PM. Expression analysis and identification of antimicrobial peptide transcripts from six North American frog species. Dis Aquat Organ. 2013;104(3):225–36. Epub 2013/06/14. 10.3354/dao02601 . 23759560

[39] Tomas ME, Kundrapu S, Thota P, Sunkesula VC, Cadnum JL, Mana TS, et al Contamination of Health Care Personnel During Removal of Personal Protective Equipment. JAMA Intern Med. 2015;175(12):1904–10. Epub 2015/10/13. 10.1001/jamainternmed.2015.4535 . 26457544

[40] Birnbach DJ, Rosen LF, Fitzpatrick M, Carling P, Arheart KL, Munoz-Price LS. Double gloves: a randomized trial to evaluate a simple strategy to reduce contamination in the operating room. Anesth Analg. 2015;120(4):848–52. Epub 2014/05/20. 10.1213/ANE.0000000000000230 . 24836472

[41] Reitzel R, Rosenblatt J, Jiang Y, Hachem R, Raad I. Disposable gendine antimicrobial gloves for preventing transmission of pathogens in health care settings. Am J Infect Control. 2014;42(1):55–9. 10.1016/j.ajic.2013.07.005. 24388469

[42] Oh JK, Rapisand W, Zhang M, Yegin Y, Min Y, Castillo A, et al Surface modification of food processing and handling gloves for enhanced food safety and hygiene. J Food Eng. 2016;187:82–91. 10.1016/j.jfoodeng.2016.04.018.

[43] Cashins SD, Alford RA, Skerrati LF. Individual study: Lethal effects of latex, nitrile, and vinyl gloves on tadpoles. Herpetol Rev. 2008;39:298–301.

[44] Pessier AP, Mendelson JR, editors. Manual for Control of Infectious Diseases in Amphibian Survival Assurance Colonies and Reintroduction Programs. IUCN/SSC Conservation Breeding Specialist Group; 2017; Apple Valley, MN.

[45] Wobeser GA. Essentials of Disease in Wild Animals. Ames, Iowa: Wiley-Blackwell Publishing; 2006. 256 p.

[46] Harp EM, Petranka JW. Ranavirus in wood frogs (*Rana sylvatica*): potential sources of transmission within and between ponds. J Wildl Dis. 2006;42(2):307–18. Epub 2006/07/28. 10.7589/0090-3558-42.2.307 . 16870853

[47] Brunner JL, Beaty L, Guitard A, Russell D. Heterogeneities in the infection process drive ranavirus transmission. Ecology. 2017;98(2):576–82. 10.1002/ecy.1644 27859036

[48] Johnson AF, Brunner JL. Persistence of an amphibian ranavirus in aquatic communities. Dis Aquat Organ. 2014;111(2):129–38. Epub 2014/10/01. 10.3354/dao02774 . 25266900

[49] Munro J, Bayley AE, McPherson NJ, Feist SW. Survival of Frog Virus 3 in Freshwater and Sediment from an English Lake. J Wildl Dis. 2016;52(1):138–42. 10.7589/2015-02-033 . 26555105

